# Root resorption followed by orthodontic treatment in individuals with anterior open bite. A complete vision focused on different treatment alternatives: A review

**DOI:** 10.21142/2523-2754-1003-2022-118

**Published:** 2022-09-28

**Authors:** Ruth Elizabeth Ramirez-Diaz, Karen Moscoso-Sivirichi, Michella Consoli-Senno

**Affiliations:** 1 School of Dentistry, San Martin de Porres University, Lima, Peru. eli_ruth@hotmail.com Universidad de San Martín de Porres School of Dentistry San Martin de Porres University Lima Peru eli_ruth@hotmail.com; 2 School of Dentistry, Alas Peruanas University, Lima, Peru. karenmsivirichi@gmail.com Universidad Alas Peruanas School of Dentistry Alas Peruanas University Lima Peru karenmsivirichi@gmail.com; 3 School of Dentistry, Cayetano Heredia University, Lima, Peru. michella.vcs4@gmail.com Universidad Peruana Cayetano Heredia School of Dentistry Cayetano Heredia University Lima Peru michella.vcs4@gmail.com

**Keywords:** open bite treatment, incisor root resorption, molar root resorption, root resorption, aligners, tratamiento de mordida abierta, reabsorción de la raíz del incisivo, reabsorción radicular molar, reabsorción radicular, alineadores

## Abstract

The aim of this review was to determine the incidence of different types of treatment and the prevalence of root resorption in incisors induced by orthodontic treatment in patients with open bite. Libraries and electronic databases were searched, with 322 articles being selected and 55 articles considered regarding PRISMA checklist. It has been shown that apical root resorption of the incisors is more frequent in patients with premolar extractions than in those treated without extractions, due to greater apical displacement during retraction of the anterior teeth in the space closure phase. On the other hand, it has been described that intrusion of posterior teeth is four times more likely to cause root resorption than extrusion movement, thereby increasing the risk of root resorption in posterior teeth compared to conventional orthodontic treatment not requiring molar intrusions. Finally, aligners, such as orthodontic treatments with fixed appliances, have not been shown to induce clinically significant root resorption in open bite individuals. Literature on root resorption in open bite treatments is scarce making difficult conclusions difficult. However, the amount of root loss in cases of open bite seems to be similar to that of individuals without open bite.

## RESUMEN

El objetivo de esta revisión fue determinar la incidencia de los diferentes tipos de tratamiento y la prevalencia de la reabsorción radicular en los incisivos inducida por el tratamiento de ortodoncia en pacientes con mordida abierta. Se realizaron búsquedas en bibliotecas y bases de datos electrónicas, se seleccionaron 322 artículos y se consideraron 55 artículos de acuerdo con las guías PRISMA. Se ha demostrado que la reabsorción radicular apical de los incisivos es más frecuente en pacientes con extracciones de premolares que en los tratados sin extracciones, debido al mayor desplazamiento apical durante la retracción de los dientes anteriores en la fase de cierre de espacios. Por otro lado, se ha descrito que la intrusión de los dientes posteriores tiene cuatro veces más probabilidades de causar reabsorción radicular que el movimiento de extrusión, lo que aumenta el riesgo de reabsorción radicular en los dientes posteriores en comparación con el tratamiento de ortodoncia convencional que no requiere intrusiones molares. Finalmente, no se ha demostrado que los alineadores, como los tratamientos de ortodoncia con aparatos fijos, induzcan una reabsorción radicular clínicamente significativa en individuos con mordida abierta. La literatura sobre reabsorción radicular en tratamientos de mordida abierta es escasa, lo que dificulta conclusiones definitivas. Sin embargo, la cantidad de pérdida de raíz en los casos de mordida abierta parece ser similar a la de las personas sin mordida abierta.

## INTRODUCTION

External apical resorption (EAR) is one of the main undesired effects that may occur in orthodontic treatment. Several factors can favor EAR, including the magnitude, direction, time of use and type of orthodontic force. The risk of EAR has been calculated as being 3.72-fold higher in patients with premolar extractions than in those without extraction. However, in general, the origin of EAR is multifactorial and is not only related to orthodontic treatment ^1^^-^^15^. Other factors identified include genetic and systemic factors, sex, and the type of malocclusion ^16^^-^^18^.

Treatment of anterior open bite is considered a challenge for many orthodontists, with a prevalence ranging between 1.5% and 11% in different age groups and populations ^19^^-^^21^. In addition, it is associated with mouth breathing, sucking habits, and altered development of the mandible and maxilla, and thus, orthodontic treatment must be interdisciplinary, considering several treatment alternatives including interceptive orthodontics, orthodontic camouflage, mini-implants, mini-plates and orthodontic-surgical treatment ^22^^-^^24^.

It is important to recognize the magnitude of root resorption in patients with anterior open bite treated orthodontically, since its development is frequent after performing biomechanics to treat this condition with posterior intrusion and inevitable anterior extrusion ^25^^-^^27^. However, although it has not been clearly demonstrated that anterior extrusive orthodontic movement induces greater root resorption ^26^^,^^28^^-^^30^, several studies have related root loss after extrusive movement to a history of trauma and inflammatory processes ^31^^,^^32^. Root resorption is an undesired risk that must be detected as soon as possible, with adequate periodic radiological monitoring and the corresponding therapeutic precautions ^33^^,^^34^. Additionally, it is necessary to clarify the magnitude of root resorption in the different open bite treatment alternatives and whether it represents an oral health risk that should be considered. Therefore, the aim of this literature review was to compare root resorption of maxillary incisors according to the different alternatives of orthodontic treatment for correcting anterior open bite.

## METHODOLOGY

### Information sources and search strategy

This review included a bibliographic search in the main sources of scientific information, including Medline via PubMed, Scopus and the Cochrane library. The search was carried out until May 5, 2022, and the search strategies are presented in Table 1. 

**Table 1:** Strategies in the search for scientific articles in the main sources of information

Source	Search Items
PubMed (89)	(((root resorption) OR (dental resorption)) OR (orthodontic dental resorption)) AND ((open bite) OR (open bite treatment))
Scopus (228)	(TITLE-ABS-KEY (root AND resorption OR dental AND resorption OR incisor AND root AND resorption OR molar AND root AND resorption)) AND (open AND bite AND treatment OR open AND bite)
SciELO (2)	(root resorption OR dental resorption OR incisor root resorption OR molar root resorption) AND (open bite OR open bite treatment)

### Study selection

**Fig 1:**
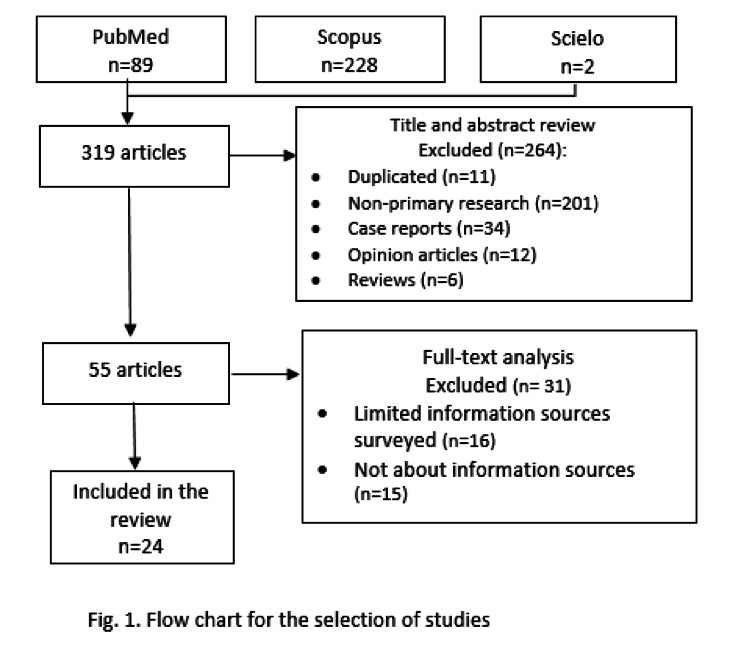
Flow chart for the selection of studies.

Finally, 24 papers meeting the following inclusion criteria were included: observational studies, and randomized clinical trials in humans of both sexes. *In vitro* studies, letters to the editor, personal opinions and case reports were excluded. (Fig. 1).

## RESULTS AND DISCUSSION

### Orthodontic treatment of open bite with and without premolar extractions

Six articles met the inclusion criteria. All were studies published in journals indexed in reliable sources of information and described that patient with premolar extractions present more apical root resorption of the incisor than patients treated without extractions, due to greater apical displacement during the retraction of the anterior teeth in the spaces closing phase. McNab *et al*.^35^ found that the amount of root resorption was 3.72-fold greater in patients with extractions than in those without (95% confidence interval: 1.96-7.04). It should be noted that the studies evaluated significantly differed in terms of the study design used, the methodology, the comparison with a control group, the characteristics of the treatment, the radiographic technique used, the standardization of the capture and, therefore, generally had a small sample size ^16^^,^^35^^-^^39^. In addition, several studies did not distinguish the biological variables related to the patient and the characteristics of each treatment. All these variables can directly influence the degree of root resorption of the teeth evaluated and are common problems that do not allow generalization of the results, thereby limiting their external validity ^16^^,^^37^^-^^39^.

On the other hand, other studies showed that the treatment of open bite carried out with premolar extractions did not show statistically significant differences in reference to the degree of root resorption when compared with groups treated with extractions, but with normal overbite. Likewise, they found no differences when comparing groups with open bite treated without extractions versus groups with normal overbite treated without extractions. Overall, there was a statistically significant correlation between root resorption and change in horizontal overjet in premolar extraction groups, suggesting that incisor retraction could be considered a predictive factor of greater root resorption ^16^. This demonstrates that it is not the initial condition of open bite that generates a greater risk of root resorption but rather root resorption is due to retraction of the incisors as a result of the retraction movement in cases treated with dental extractions ^36^.

Regarding the use of anterior vertical elastics used for open bite closure and treatment time, neither showed a significant association or correlation with the degree of root resorption. This is important since it allows clinicians to more adequately manage these cases ^16^^,^^37^.

It is concluded that there is a significant difference in root resorption in orthodontic treatments in patients with open bite with and without extractions, with cases treated with extractions presenting a greater amount of root resorption following retraction of the anterior segment for closure of spaces.

### Orthodontic treatment of open bite with intrusion of molars and premolars

In relation to this topic, 13 articles met the selection criteria. Several therapeutic alternatives are able to achieve intrusion of the posterior teeth, including traditional biomechanical techniques, such as headgear, chin rest, and active vertical corrector with magnets, among others. However, these require a high level of cooperation on the part of the patient to effectively control molar intrusion ^1^^,^^37^^,^^38^. Likewise, the Begg technique, which includes tip-back bends mesial to the tube of first molars, seeks to achieve intrusive effects similar to the Bioprogressive technique ^38^^-^^41^. In recent years, skeletal anchorage devices, such as miniplates and miniscrews, have gained popularity due to their great ability to provide stable anchorage throughout orthodontic treatment and the low need for patient collaboration. However, the secondary effects of intrusion with the use of any of these methods have been little studied. Nonetheless, the few studies available allow some conclusions to be drawn.

Anterior open bite is characterized by maxillary and/or mandibular vertical dentoalveolar excess and hyperdivergent maxillomandibular growth, making its treatment a clinical challenge for orthodontists ^42^^-^^45^. Indeed, to solve this vertical alteration, orthodontists plan a true molar intrusion that corrects the skeletal open bite. In this sense, it has been reported that the intrusion of posterior teeth is four times more likely to cause root resorption than extrusion movement, thereby increasing the risk of root resorption in posterior teeth compared to conventional orthodontic treatment that does not require molar intrusions ^46^.

Some studies described that apical root resorption in posterior teeth subjected to intrusive mechanics ranges from 0.02 mm to 2.49 mm while in other studies resorption ranged from 0.84 mm to 1.00 mm, with forces between 200 g and 400 g. However, it is important to note that this amount of resorption, although present and clinically undesirable, is not very relevant and does not place the dental health of patients at risk ^25^^,^^47^.

Some researchers recommend the use of cone beam computed tomography to detect the presence of root resorption, since conventional radiographs such as lateral cephalogram, panoramic and periapical films are not sufficiently precise. Neither are they the indicated methods to evaluate the amount of resorption, due to errors of measurement, magnification and overlaps, which can lead to underestimation or overestimation of the amount of root resorption ^10^^,^^16^^,^^25^^,^^47^^,^^48^.

Taking into account the few studies available and the unpredictable methods used, such as conventional radiographs, it cannot be concluded that there is significant apical resorption after performing molar and premolar intrusion mechanics. Nonetheless, it should be kept in mind that the smaller the force, that is, light and continuous forces, the fewer side effects intrusion mechanics will have ^25^^,^^47^^,^^48^.

## TREATMENT OF OPEN BITE WITH ALIGNERS

Regarding this topic, 5 articles meeting the selection criteria were found, indicating that aligners are an orthodontic therapeutic alternative and are promoted as an aesthetic alternative compared to fixed appliances. Aligners were initially indicated in cases of low complexity, without skeletal discrepancies, mainly with mild crowding, favoring the low incidence and severity of root resorption by the creation of intermittent forces due to the occasional extraction of the aligner during the consumption of food and maintenance of hygiene. However, the use of aligners has evolved rapidly, and new built-in features facilitate the treatment of more complex malocclusions ^49^^-^^51^.

There is at least one report of root resorption in the general treatment of malocclusions with aligners. This indicates that as with other orthodontic treatments with fixed appliances, the incidence of root resorption is not reduced. In other studies, post-treatment cases with fixed appliances and aligners presented 85.3% and 41.81% of root resorption, respectively, mainly affecting the upper lateral incisors and the lower lateral and central incisors ^51^^-^^53^.

Although aligners have often been considered of limited efficacy, some studies have demonstrated their success in the treatment of mild anterior open bites, producing a bite block effect and maintaining vertical control, and thereby making aligners a possible alternative treatment for open bite cases ^49^^,^^54^.

Few studies have described the use of aligners for the treatment of open bite and the effect on incisors, concluding that incisor root resorption was less than 1 mm, which is considered a clinically insignificant value. Moreover, the results cannot be extrapolated because of the few studies available and, thus, further studies are necessary to clarify the possible benefits of aligners in the treatment of open bite ^55^.

## LIMITATIONS

Although this study provides valuable information for dentistry, the results must be considered with caution because of the moderate risk of bias of the studies included. Likewise, the external validity does not allow generalization of the results.

## CONCLUSIONS

Root resorption of incisors after orthodontic treatment of patients with open bite seems to be similar to that of patients with normal overbite and should be taken into account in the clinical practice of orthodontists.

While open bite does not seem to increase root resorption, cases treated with extractions present a greater amount of root resorption in the anterior teeth after incisor retraction for space closure compared to cases without extractions.

Skeletal anchorage devices provide greater stability, control and efficacy in the mechanics of treatment of molar intrusion in addition to not requiring patient collaboration. However, intrusion of molars does not significantly affect the dental roots, recommending the use of light and continuous forces.

Root resorption of the upper incisors after orthodontic movement by intrusion or extrusion occurs in patients with a history of short roots and/or root abnormalities and in those in whom the force applied during orthodontic treatment exceeds the repair capacity of the cement in intensity and duration.

## Financing Sources:

Self-financed
